# Dexmedetomidine as adjunct treatment for severe alcohol withdrawal in the ICU

**DOI:** 10.1186/2110-5820-2-12

**Published:** 2012-05-23

**Authors:** Samuel G Rayner, Craig R Weinert, Helen Peng, Stacy Jepsen, Alain F Broccard

**Affiliations:** 1 University of Minnesota Medical School, 1803 E John Street Seattle, Seattle, WA 98112, USA; 2 Division of Pulmonary, Allergy, and Sleep Medicine; Fairview-Southdale Hospital, University of Minnesota, Minneapolis, MN, USA; 3 Fairview-Southdale Hospital, 6401 France Ave. S., Edina, MN, 55435, USA; 4 Division of Pulmonary, Allergy, and Sleep Medicine; Fairview-Southdale HospitalUniversity of Minnesota, University of Minnesota, Minneapolis, MN, USA; 5 Fairview-Southdale Hospital, 6401 France Ave. S. Edina, Minnesota, MN, 55435, USA

**Keywords:** Alcohol withdrawal delirium, Alcohol withdrawal syndrome, Dexmedetomidine, Intensive care, Critical care, Benzodiazepines

## Abstract

**Background:**

Patients undergoing alcohol withdrawal in the intensive care unit (ICU) often require escalating doses of benzodiazepines and not uncommonly require intubation and mechanical ventilation for airway protection. This may lead to complications and prolonged ICU stays. Experimental studies and single case reports suggest the α_2_-agonist dexmedetomidine is effective in managing the autonomic symptoms seen with alcohol withdrawal. We report a retrospective analysis of 20 ICU patients treated with dexmedetomidine for benzodiazepine-refractory alcohol withdrawal.

**Methods:**

Records from a 23-bed mixed medical-surgical ICU were abstracted from November 2008 to November 2010 for patients who received dexmedetomidine for alcohol withdrawal. The main analysis compared alcohol withdrawal severity scores and medication doses for 24 h before dexmedetomidine therapy with values during the first 24 h of dexmedetomidine therapy.

**Results:**

There was a 61.5% reduction in benzodiazepine dosing after initiation of dexmedetomidine (n = 17; *p <* 0*.*001) and a 21.1% reduction in alcohol withdrawal severity score (n = 11; *p* = .015). Patients experienced less tachycardia and systolic hypertension following dexmedetomidine initiation. One patient out of 20 required intubation. A serious adverse effect occurred in one patient, in whom dexmedetomidine was discontinued for two 9-second asystolic pauses noted on telemetry.

**Conclusions:**

This observational study suggests that dexmedetomidine therapy for severe alcohol withdrawal is associated with substantially reduced benzodiazepine dosing, a decrease in alcohol withdrawal scoring and blunted hyperadrenergic cardiovascular response to ethanol abstinence. In this series, there was a low rate of mechanical ventilation associated with the above strategy. One of 20 patients suffered two 9-second asystolic pauses, which did not recur after dexmedetomidine discontinuation. Prospective trials are warranted to compare adjunct treatment with dexmedetomidine versus standard benzodiazepine therapy.

## Background

The medical consequences of excessive alcohol use are all too familiar to clinicians. Alcohol abuse and dependence have a combined prevalence of 7.4–9.7% in the United States [1]. This increases to 10–33% in intensive care unit (ICU) patients; 18% of these patients develop acute withdrawal symptoms during their hospitalization [2]. Alcohol withdrawal syndrome (AWS) is an abstinence syndrome characterized by autonomic hyperactivity, hallucinations, and seizures, termed alcohol withdrawal delirium or delirium tremens when accompanied by a persistent altered sensorium and severe hyperadrenergic state. This severe form of withdrawal develops in approximately 5% of patients who abruptly cease ethanol ingestion, often during an acute medical illness, and generally begins within 48–96 h after cessation of alcohol intake [3-5].

Chronic ethanol ingestion leads to changes in the expression patterns of inhibitory γ-aminobutyric acid (GABA) receptors within the central nervous system (CNS) and inhibition of the excitatory glutamate system. With cessation of alcohol intake, disrupted GABA signaling and rebound increase in glutamate signaling produce CNS hyperexcitation leading to the clinical manifestations of alcohol withdrawal [6-8]. Increases in noradrenergic and dopaminergic signaling may play a role in autonomic instability and alcoholic hallucinations, respectively [3,9,10]. Benzodiazepines, which act through GABA receptors, are logical interventions for abstinence-associated CNS excitation, and reduce symptom duration, delirium, incidence of seizure and, in trials comparing benzodiazepines with neuroleptics, mortality [4,11,12]. In severe AWS, however, benzodiazepine monotherapy may be less effective. Patients with significant tolerance to alcohol exhibit cross-tolerance to the GABA-mediated effects of benzodiazepines, and the requirement for high doses of benzodiazepines can lead to oversedation, respiratory insufficiency, and worsening of delirium [4,13,14]. Large doses of benzodiazepines increase the risk of aspiration and intubation, increasing complication rates, length of stay, and hospital costs [14].

Anticonvulsants may have efficacy similar to benzodiazepines in mild-moderate alcohol withdrawal, but limited data suggest lower efficacy in AWS [15,16]. β-adrenoreceptor antagonists and the α_2_ adrenoreceptor agonist clonidine are used to reduce autonomic symptoms but have not been well studied in critical care cases, and certain agents (e.g., propranolol) may worsen delirium [4,15,17]. Propofol, which acts through both GABAergic and glutamatergic pathways in the CNS, is effective in the management of benzodiazepine-refractory AWS, but the majority of patients who receive propofol require intubation during treatment [18,19]. Propofol therapy also causes hypertriglyceridemia, hypotension, and is associated with the propofol infusion syndrome [18].

The α_2_ agonist, dexmedetomidine, is indicated for sedation of mechanically ventilated patients in the ICU and procedural sedation in nonintubated patients [20,21]. It has shown efficacy in murine models and single human case reports of alcohol withdrawal [22-25]. There also are reports of dexmedetomidine for nonethanol or poly-agent withdrawal syndromes [26,27]. We report our experience using dexmedetomidine for benzodiazepine-refractory AWS in a single ICU.

## Methods

### Treatment information

This study was approved by the University of Minnesota Human Subjects Protection Program (approval code number 1101 M94834) and a waiver of informed consent for data abstraction was granted for patients admitted to the open 23-bed medical-surgical-neuroscience ICU at the Fairview-Southdale Hospital. Treatment of patients with alcohol withdrawal symptoms involves standardized physician orders, including vitamin, electrolyte, and intravenous fluid replacement therapy. Additionally, a nursing-administered alcohol withdrawal severity assessment protocol is utilized, with ten parameters consisting of vital signs and physical and behavioral findings, which are assessed and assigned a point value from 1–3 [28]. Ranges of total points are linked to dose ranges of intravenous or oral lorazepam that nursing personnel can administer, from none for a total score below 4, to a maximum range of 2–4 mg for a score above 10. Frequency of withdrawal scoring depends on prior score and route of lorazepam administration, with a minimum of every 4 h and a maximum of every 10 min for intravenous lorazepam therapy or every hour for oral lorazepam therapy. Standing orders may include 2 mg of intravenous haloperidol per hour for severe agitation or hallucinations. This protocol may be initiated on the general wards, with transfer to the ICU based on clinical judgment and need for monitoring during high-dose benzodiazepine therapy. Dexmedetomidine is not administered on the general wards. Any decision to initiate dexmedetomidine is made by board-certified intensivists, and there is no specific protocol regarding dexmedetomidine therapy for alcohol withdrawal.

### Sample selection

The electronic medical record was reviewed for ICU patients from November 2008 to November 2010 who received ICD-9 codes consistent with alcohol withdrawal during hospitalization. These medical records were examined for patients who received dexmedetomidine during their ICU stay, and clinician documentation was examined to ensure that dexmedetomidine was received solely for the management of alcohol withdrawal symptoms. Patients were excluded who had severe comorbid disease, including several with CNS trauma or cerebrovascular accidents, one with end-stage metastatic carcinoma, and one patient with severe sepsis. To reduce selection bias, all consecutive patients who met study criteria were included.

### Data collection

Data from 24 h before dexmedetomidine initiation to 24 h after were abstracted into a predefined Excel template. We abstracted hourly dose of dexmedetomidine, benzodiazepines, and haloperidol, alcohol withdrawal score, use of dexmedetomidine bolus, administration of β-blocking medications, intubation requirements, length of ICU stay, hourly systolic blood pressure and heart rate, hours spent with heart rate <60 or >100 beats per minute, and hours with systolic blood pressure <90 or >140 mm Hg. Clinician-documented adverse effects of dexmedetomidine were recorded. The few instances of diazepam or midazolam administration were converted to lorazepam dosing using factors of 0.2 and 0.5 respectively [29].

### Statistical analysis

We compared group means in the 24 h before dexmedetomidine administration with means for the first 24 h of dexmedetomidine therapy. We present the difference between these means, as well as the 95% confidence interval for this difference. Normality was checked for all variables under study using D’Agostino-Pearson testing, and paired two-tailed *t* tests were used to compare group means with *p* < 0.05 defined as significant. Because we depended on clinical documentation done during routine care, there were missing data, especially in alcohol withdrawal severity scoring where analysis was limited to the 11 patients with complete scoring data.

## Results

There were 19 male subjects and 1 female subject, with a mean age of 44.9 years (95% confidence interval (CI), 40.9-48.9). One patient required intubation and all survived to discharge. Mean length of stay in the ICU was 98.5 h (95% CI, 54.0-143.0). For the 19 nonintubated patients, mean length of dexmedetomidine therapy was 49.1 h (95% CI, 36.8-61.4) and mean dexmedetomidine dose was 0.53 μg/kg/h (95% CI, 0.44-0.62). Five patients received bolus therapy on initiation of dexmedetomidine. Three patients had less than 24 h of information available before dexmedetomidine initiation. One of these patients had 6 hours of baseline information available and was used in calculations of mean vitals but not calculations of time with vitals outside of defined ranges. The other two had almost no baseline data and were excluded from analysis. One patient was intubated 13 h after dexmedetomidine initiation and was not included in analyses of medication dosing or vitals, because, once intubated, he received additional sedative and analgesic agents based on a separate nursing-driven order set for ventilated patients. Finally, all other patients were on dexmedetomidine for at least 24 h, except for one who was on dexmedetomidine for a total of 14 h. This patient’s data were extrapolated to 24 h for statistical analysis. The sample sizes for separate analyses are reported below. D’Agostino-Pearson testing demonstrated normality for all variables under study (*p* > 0.1).

### Benzodiazepine and haloperidol dosing

**Figure 1:**
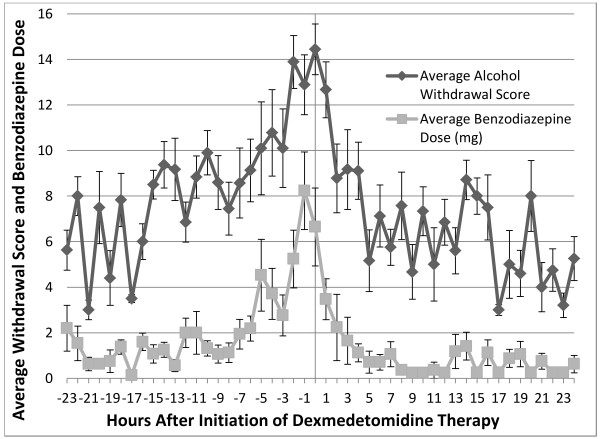
**Average alcohol withdrawal scoring and average benzodiazepine dose (mg) versus initiation of dexmedetomidine therapy.** Alcohol withdrawal scores were averaged each hour for all patients with complete data available (n = 11) as was average hourly benzodiazepine dose in mg (n = 17). Measurements were recorded from 24 h prior to dexmedetomidine therapy through the first 24 h on dexmedetomidine. Negative numbers represent data prior to the initiation of the study drug and the time point 0 represents the initiation of dexmedetomidine. Error bars reflect the standard error around each mean value.

Seventeen patients were available for analysis of benzodiazepine dosing. A statistically significant mean 32.4 mg (61.5%) per 24 h decrease in benzodiazepine dose (*p* < 0.001; 95% CI, 16.7-48.1) was administered in the 24 h period following dexmedetomidine initiation (Table 1). Whereas patients often received some oral benzodiazepine therapy upon initial presentation, most medication was provided intravenously. Figure 1 shows that the escalating dose of benzodiazepines rapidly decreases after initiation of dexmedetomidine. Mean haloperidol dosing in the same 17 patients decreased by 5.6 mg (46.7%) per 24 h (*p* = 0.052; 95% CI, −0.03–11.23) following initiation of dexmedetomidine (Table 1).

**Table 1:** Analysis Comparing Pre- and Post-Dexmedetomidine Data

**Endpoint**	**Sample size (n) for endpoint**	**24 h before dex**	**First 24 H of dex therapy**	**Decrease in values following dex initiation (percent decrease)**	***p*value**	**95% Confidence interval for decrease seen after dex initiation**
Average Alcohol Withdrawal Scoring	11	9.0	7.1	1.9 (21.1%)	0.015	0.44–3.36(4.9%–37.3%)
Average Benzodiazepines Received (mg)	17	52.7	20.3	32.4 (61.5%)	<0.001	16.7–48.1(31.7%–91.3%)
Average Haloperidol Received (mg)	17	12.0	6.4	5.6 (46.7%)	0.052	0.03–11.23(−0.36–93.6%)
Average HR	17	102.8	79.3	23.4 (22.8%)	<0.001	18.4–28.4(17.9%–27.6%)
Average SBP	17	140.2	126.7	13.5 (9.6%)	0.002	5.32–21.68(3.8%–15.4%)
Hours With HR > 100	16	13.3	2.3	10.9 (82.0%)	<0.001	7.4–14.4(55.6–108.3%)
Hours With SBP >140	16	11.0	6.3	4.7 (42.3%)	0.02	0.8–8.6(3.8–15.4%)
Hours With HR <60	16	0.0	2.0	−2.0	0.055	4.05–0.05
Hours With SBP <90	16	0.0	0.9	−0.9	0.079	1.89–0.09

For dosing, total dose per 24 h period for each patient was averaged. For average vitals, mean values per 24 h period for each patient were averaged. For vitals outside normal range, total time outside normal range was calculated for each patient per 24 h period, and averaged. A sample size of 16 (instead of 17) was used for analyses of time spent with vitals outside normal, due to one patient having only six hours of available data. P values obtained from two-tailed paired t-tests. 95% confidence interval was calculated using t distribution for a paired sample and n-1 degrees of freedom. Dex = dexmedetomidine; SBP = systolic blood pressure; HR = heart rate.

### Alcohol withdrawal scoring

Complete data for 11 patients were analyzed for alcohol withdrawal scoring. There was a mean decrease of 1.9 points (21.1%) on the alcohol withdrawal severity scale (p < 0.015; 95% CI .44-3.36) in the 24 h period following dexmedetomidine initiation (Table 1). Due to the sample size for this variable, non-parametric analysis with two-tailed Wilcoxon matched pairs testing was also done and significant (p < 0.03). Figure 1 shows a rapid decrease in the score within the first 4 h after initiation of dexmedetomidine with a slower decline over the next 20 h.

### Heart rate and administration of β-blocking medications

A sample size of 17 patients was used for mean heart rate analysis. There was a statistically significant decrease in heart rate by 23.4 beats per minute (22.8%) in the 24 h following dexmedetomidine initiation (*p* < 0.001; 95% CI, 18.4-28.4). In a sample size of 16, there was a statistically significant decrease of 10.9 h (82%) spent with heart rate >100 beats per minute (*p* < 0.001; 95% CI, 7.4-14.4) in the 24 h after dexmedetomidine initiation (Table 1). Of the 20 study patients, 4 were continued on their outpatient β-blockers while in the ICU. Additionally, during their ICU stays two patients received oral metoprolol once, one patient received oral metoprolol twice, one patient was given IV labetalol twice, and one patient received both oral metoprolol and IV labetalol occasionally.

### Blood pressure

**Figure 2:**
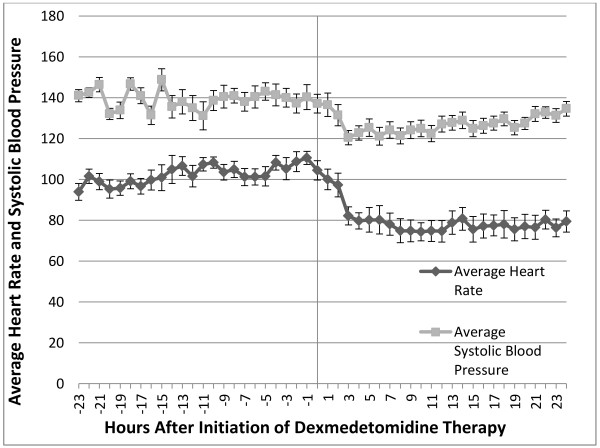
**Hourly systolic blood pressure and heart rate versus initiation of dexmedetomidine therapy.** Systolic blood pressure and heart rate values were averaged each hour for all 16 patients with data available. Measurements were recorded from 24 h prior to dexmedetomidine therapy through the first 24 h on dexmedetomidine. Negative numbers represent data prior to the initiation of the study drug and the time point 0 represents the initiation of dexmedetomidine. Error bars reflect the standard error around each mean value.

A sample size of 17 patients was used for analysis of mean systolic blood pressure. There was a statistically significant decrease in systolic blood pressure of 13.5 mm Hg (9.6%) following dexmedetomidine initiation (*p* = 0.002; 95% CI, 3.8-15.4%). In a sample size of 16, there was a statistically significant decrease of 4.7 h (42.3%) in time with systolic blood pressure above 140 mm Hg (*p* = 0.02; 95% CI, 0.8-8.6) in the 24 h following dexmedetomidine initiation (Table 1). Figure 2 shows that previously elevated heart rate and blood pressure decrease sharply during the first 2 hours after initiation of dexmedetomidine.

### Adverse outcomes

One patient out of 20 was intubated for respiratory failure. Significant adverse effects related to dexmedetomidine therapy were suspected in only one patient, in whom dexmedetomidine was discontinued due to two 9-second asystolic pauses. These were noted on telemetry and did not require specific treatment. No further abnormalities in heart rate/rhythm were noted following discontinuation of dexmedetomidine. Finally, there were nonstatistically significant increases in bradycardia (<60 beats per minute) and systolic hypotension (<90 mm Hg) with *p* values of 0.055 and 0.079 respectively. These patients did not suffer any instances of heart rate below 50 or systolic blood pressure below 80.

## Discussion

In this study, dexmedetomidine use in patients with severe alcohol withdrawal symptoms led to rapid and statistically significant reductions in benzodiazepine dosing, alcohol withdrawal score, tachycardia, and systolic hypertension. Most effects occurred within 4 h of dexmedetomidine initiation (with bolus dose at initiation used in only 5 patients) and were sustained over 24 h. Dexmedetomidine rates less than 0.7 μg/kg/h were generally sufficient. Intubation was required only in one patient who, importantly, had received the highest amount of lorazepam before dexmedetomidine initiation of any patient. The only adverse effect that required discontinuation of therapy was occurrence of two 9-second asystolic pauses in one patient, which resolved with discontinuation of dexmedetomidine. This patient received an initial bolus of dexmedetomidine of 1 μg/kg and a high average infusion rate of 0.75 μg/kg/h, both of which are associated in the literature with symptomatic bradycardia [21]. There were nonsignificant increases in mild hypotension and bradycardia (systolic blood pressure 80–90 and heart rate 50–60, respectively) following dexmedetomidine initiation.

Our study adds to growing literature on the potential use of dexmedetomidine for AWS. Studies performed in mice found that dexmedetomidine reduced rigidity, tremor, and irritability during ethanol withdrawal, while exhibiting neuroprotective effects and potential antiepileptic activity [22,23,30]. Two case reports support the use of dexmedetomidine in benzodiazepine-refractory AWS [24,25]. Dexmedetomidine also was applied successfully in patients undergoing sedative/analgesic or polysubstance withdrawal in two case series consisting of two and three patients, respectively [26,27].

Dexmedetomidine has the ability to produce arousable sedation without respiratory depression [21,31,32]. Through actions on α_2_ receptors dexmedetomidine leads to sedation, analgesia, inhibition of the sympathetic nervous system, and increased cardiac vagal tone [33,34]. These characteristics make dexmedetomidine attractive for treatment of the hyperadrenergic state seen during alcohol withdrawal. Dexmedetomidine has advantages over midazolam and lorazepam in providing sedation with minimal delirium and over haloperidol in managing intubated delirious patients [35-37]. Additional advantages of dexmedetomidine are its rapid onset and relatively short half-life, as well as its favorable side effect profile [38]. Pharmacologically, dexmedetomidine has advantages over the α_2_ agonist clonidine given its greater titratability (half-life of 2.3 h versus 6–10 h for clonidine) and its eightfold greater selectivity for the α_2_ receptor, which is thought to be involved in centrally mediated anxiolysis and sedation [34,39]. The use of dexmedetomidine does have risks of hypotension and bradycardia, although a recent meta-analysis found no difference in rates of hypotension requiring intervention between dexmedetomidine and other sedatives, and found a risk of significant bradycardia only when a loading dose or rates over 0.7 μg/kg/h were employed [20,21]. Continuous cardiac monitoring is advised and care should be taken in patients with advanced heart block, severe ventricular dysfunction, hypovolemia, or when receiving other vasodilators or negative chronotropic agents. Dose reduction may be necessary in patients with hepatic dysfunction. For further information regarding use, dosing, indications, and adverse effects, we refer the reader to a recent review of this medication as well as the manufacturer’s website [39,40].

Our study is limited by its retrospective nature and lack of a control group. Additionally, our ICU does not have a protocol specifying when dexmedetomidine should be added to benzodiazepine therapy; therefore, dexmedetomidine was likely started at different points in the alcohol withdrawal process as clinicians’ thresholds for determining that benzodiazepine therapy had “failed” may differ. One limitation of dexmedetomidine therapy itself is that it may not have the ability to prevent alcohol withdrawal seizures. Whereas laboratory/rodent models provide some support for α_2_ agonists as anticonvulsants, an older study comparing clonidine with traditional therapy for alcohol withdrawal seizures was not promising, and dexmedetomidine cannot currently be recommended as monotherapy for AWS [22,41].

## Conclusions

The ability to provide sedation and reduce autonomic hyperactivity with potentially less respiratory distress and delirium than seen with benzodiazepine therapy makes dexmedetomidine an attractive adjunct medication for the treatment of severe alcohol withdrawal. It is especially appealing when symptoms are refractory to high doses of benzodiazepines, because it acts through a GABA-independent pathway. In this study, adjunct therapy with dexmedetomidine in severe alcohol withdrawal patients poorly controlled on, or experiencing significant adverse effects with, traditional therapy led to reductions in benzodiazepine dosing, a decrease in alcohol withdrawal scoring, and decreases in heart rate and blood pressure. The uncontrolled, retrospective nature of our study precludes definite conclusions about the efficacy or safety of dexmedetomidine for severe alcohol withdrawal. At this time, a prospective, controlled study is indicated to help determine whether dexmedetomidine as adjunct therapy for severe alcohol withdrawal truly leads to clinically meaningful outcomes, such as decreased intubation rates, decreased complication rates, or decreased length of ICU stay. Once such a trial has been performed, further studies can be done in patients undergoing severe alcohol withdrawal to help precisely define the indications, dosing schedule, and timing for this promising therapy.

## Competing interests

The authors report no receipt of funding for this study and no potential conflicts of interest with any companies or services whose products are discussed in this article.

## Authors’ contributions

SGR was the lead author of this study and contributed to study design, data collection, statistical analysis, and manuscript preparation/review. He also guarantees, to the best of his knowledge, the factual and statistical validity of the information contained herein. CRW contributed to study design, application for IRB approval, and manuscript preparation and review. HP contributed to patient selection and manuscript review. SJ contributed to study design and manuscript review. AFB was the principal investigator of this study and involved in oversight of study design, data collection and analysis, and manuscript preparation/review. All authors read and approved the final manuscript.
